# A Note on the Prevention of Pediculosis

**Published:** 1918-01

**Authors:** 


***A Note on the Prevention of Pediculosis.*** By Capt. J. A. Gunn, R. A. M. C. T. Abs. from the *Brit. Med. Jour.*, May 5, 1917, p. 579.

Since experience proved that simple cleanliness was not enough to prevent pediculosis, the author, two years ago, began the practice of dipping underclothing in a parasiticide solution, which, he states, can be easily and cheaply made by adding an ounce and a half each of naphthaline and sulphur to one gallon of benzol or petrol. This treatment has been used especially with underclothing made of a thin, light, cheap material, called butter-muslin, and on the whole has proved inexpensive and unobjectionable. Such garments, already dipped, have been distributed as a gift to men and officers for two years, and they are unanimously declared to have afforded complete protection against the body louse. Since this experiment has been made as a charitable entreprise, it has been limited in extent, and the author has been unable to get complete first-hand data regarding the results. What is positively known, however, leads him to believe that this method should be employed more generally because of its obvious advantages over powder spraying, bags, and the various other attempts to combat vermin. His method appears equally effective in preventing scabies.

